# A first-generation microsatellite-based genetic linkage map of the Siberian jay (*Perisoreus infaustus*): insights into avian genome evolution

**DOI:** 10.1186/1471-2164-10-1

**Published:** 2009-01-03

**Authors:** Sonja Jaari, Meng-Hua Li, Juha Merilä

**Affiliations:** 1 Ecological Genetics Research Unit, Department of Biological and Environmental Sciences, PO Box 65, FIN-00014 University of Helsinki, Finland

## Abstract

**Background:**

Genomic resources for the majority of free-living vertebrates of ecological and evolutionary importance are scarce. Therefore, linkage maps with high-density genome coverage are needed for progress in genomics of wild species. The Siberian jay (*Perisoreus infaustus; Corvidae*) is a passerine bird which has been subject to lots of research in the areas of ecology and evolutionary biology. Knowledge of its genome structure and organization is required to advance our understanding of the genetic basis of ecologically important traits in this species, as well as to provide insights into avian genome evolution.

**Results:**

We describe the first genetic linkage map of Siberian jay constructed using 117 microsatellites and a mapping pedigree of 349 animals representing five families from a natural population breeding in western Finland from the years 1975 to 2006. Markers were resolved into nine autosomal and a Z-chromosome-specific linkage group, 10 markers remaining unlinked. The best-position map with the most likely positions of all significantly linked loci had a total sex-average size of 862.8 cM, with an average interval distance of 9.69 cM. The female map covered 988.4 cM, whereas the male map covered only 774 cM. The Z-chromosome linkage group comprised six markers, three pseudoautosomal and three sex-specific loci, and spanned 10.6 cM in females and 48.9 cM in males. Eighty-one of the mapped loci could be ordered on a framework map with odds of >1000:1 covering a total size of 809.6 cM in females and 694.2 cM in males. Significant sex specific distortions towards reduced male recombination rates were revealed in the entire best-position map as well as within two autosomal linkage groups. Comparative mapping between Siberian jay and chicken anchored 22 homologous loci on 6 different linkage groups corresponding to chicken chromosomes Gga1, 2, 3, 4, 5, and Z. Quite a few cases of intra-chromosomal rearrangements within the autosomes and three cases of inter-chromosomal rearrangement between the Siberian jay autosomal linkage groups (LG1, LG2 and LG3) and the chicken sex chromosome GgaZ were observed, suggesting a conserved synteny, but changes in marker order, within autosomes during about 100 million years of avian evolution.

**Conclusion:**

The constructed linkage map represents a valuable resource for intraspecific genomics of Siberian jay, as well as for avian comparative genomic studies. Apart from providing novel insights into sex-specific recombination rates and patterns, the described maps – from a previously genomically uncharacterized superfamily (Corvidae) of passerine birds – provide new insights into avian genome evolution. In combination with high-resolution data on quantitative trait variability from the study population, they also provide a foundation for QTL-mapping studies.

## Background

Under various completed or ongoing projects, rapid progress has been attained in the generation of genomic resources for model organisms and domestic animals of medical, economic, or agricultural importance (e.g. [1-3]). However, genomic resources for the majority of free-living vertebrates of ecological and evolutionary importance are still scarce. For instance, in wild birds, development of genomic resources are still in their infancy, and only few initial efforts in linkage mapping [4-8], estimation of the extent of linkage disequilibrium [9,10], and syntenic comparison between related species [7,8,11-14] ] have been conducted. Hence, very limited information on the genome structure of wild bird species is available for further synthesis, as well as to study and characterize molecular underpinnings of phenotypic traits. Since wild passerine birds are important 'model' organisms in ecology and evolutionary biology, and in studies of life history evolution (e.g. [15]), behaviour (e.g. [16,17]) and evolutionary quantitative genetics in particular (e.g. [18-20]), knowledge of their genome structure and organization is vital to advance our understanding of the genetic basis of ecologically important traits [21].

Genetic maps constitute essential and powerful organizational tools for genomic research [22]. Among the most important applications of genetic maps in genomic analyses is in that they provide a platform to support studies utilizing or aiming to apply candidate gene approaches [21,23], QTL mapping [24], comparative genomics [25], and genome annotation [26]. However, construction of genetic linkage maps for non-model organisms is complicated by several factors [27,28]. One of the major obstacles for the construction of linkage maps in passerine birds (but see [4]) is the scarcity of informative genetic markers. Among a variety of molecular makers previously employed in linkage mapping in different organisms, microsatellite markers have often proven most useful due to their hypervariability, fast evolutionary rates, codominance, wide distribution throughout the genomes, and the relative ease with which they can be developed and genotyped using the polymerase chain reaction (PCR; e.g. [29]). While genetic maps exist in one form or another for various species, it is worth noticing that the studies are so far generally limited to domestic animals or natural populations of wild species that can easily be bred in captivity, or where sufficiently large litter sizes are being produced in natural settings and are accessible to sampling to allow the establishment of the pedigree necessary for linkage analysis [9]. Unfortunately, the characteristics that make populations practical for linkage mapping [9] are found only among a small fraction of species studied by ecologist and evolutionary biologists. Linkage maps have now been constructed in four populations of non-model animals for which long-term individual-based datasets are available (see also: [8]), and where natural long-term pedigrees (rather than experimental breeding programmes) have been used to follow the co-segregation of marker alleles [28]. Two of these mapping populations are in ungulate species (soay sheep, *Ovis aries *[24]; red deer, *Cervus elaphus *[27]) and two are in passerine birds (great reed warbler, *Acrocephalus arundinaceus *[4,6,13]; collared flycatcher, *Ficedula albicollis *[5,9,11]).

The Siberian jay (*Perisoreus infaustus*) is a passerine bird which has been subject to considerable ecological and evolutionary research during the past decades. Studies in its breeding biology [30-33], mating system [34,35], foraging behaviour [36], reproductive success [37], parental care and dispersal pattern [38,39], family structure [40], phenotypic plasticity [41] and levels of inbreeding [42] have been conducted. Thus, these previous studies form an appropriate setting in an initiative to explore the integration of genomics with the domain of ecology and evolutionary biology [43], provided that at least some basic knowledge of the species' genome can be obtained. With an access to detailed pedigrees of a Siberian jay population monitored over 30 years [40,42], as well as access to a novel set of polymorphic microsatellites developed for this species [44], construction of a linkage map is now realistic. In an evolutionary context, the chicken genome sequence [45] released recently facilitates genomic studies in other bird species by comparative genomic approaches [13,25]. Moreover, given the early divergence of avian lineages between passeriforms and galliforms (≈ 100 million years ago; [46]) and the high level of phylogenetic divergence between jays belonging to the Corvidae family and the other passerines for which linkage maps have been published (e.g. [47,48]), a linkage map of the Siberian jay may provide new insights into avian genome evolution, and thereby also to the extensive morphological, life historical and behavioural diversification within the order Passeriformes (see [47]).

The aim of this study was to develop a first-generation genetic linkage map for a wild population of Siberian jays on the basis of 117 microsatellites, including a novel set of 108 markers. To this end, a framework map was constructed to identify markers whose local relative orders were statistically well supported with an unambiguous location in the map. Since heterochiasmy has been observed in previous studies of many species (e.g. zebrafish [49]; and great reed warbler [4]), sex-specific variation in the recombination rate and the genetic map distance were also investigated. Furthermore, to provide a comparative perspective to address the evolution of genome organisation the extent of synteny and locus order conservation between Siberian jay and chicken was evaluated by a BLAST analysis against the chicken genome sequence.

## Results

### Characteristics of polymorphic microsatellites

Of the 117 microsatellites scored, six loci (SJ009, SJ046, SJ048, SJ069, SJ083 and SJ108) were assigned to the Z-chromosome by observation of complete cosegregation with sex and the observation of heterozygosity in some males but none of the females in the pedigrees. None of the markers examined appeared to be situated in W-chromosome since all the markers had alleles in the males. The number of informative meioses varied from 33 (SJ047) to 474 (CKL5) with an average of 284.63 informative meioses per locus. More details about levels of genetic variability (*H*_O_, *H*_E _and PIC) are shown in Additional File 1.

### Genetic linkage maps

**Figure 1:**
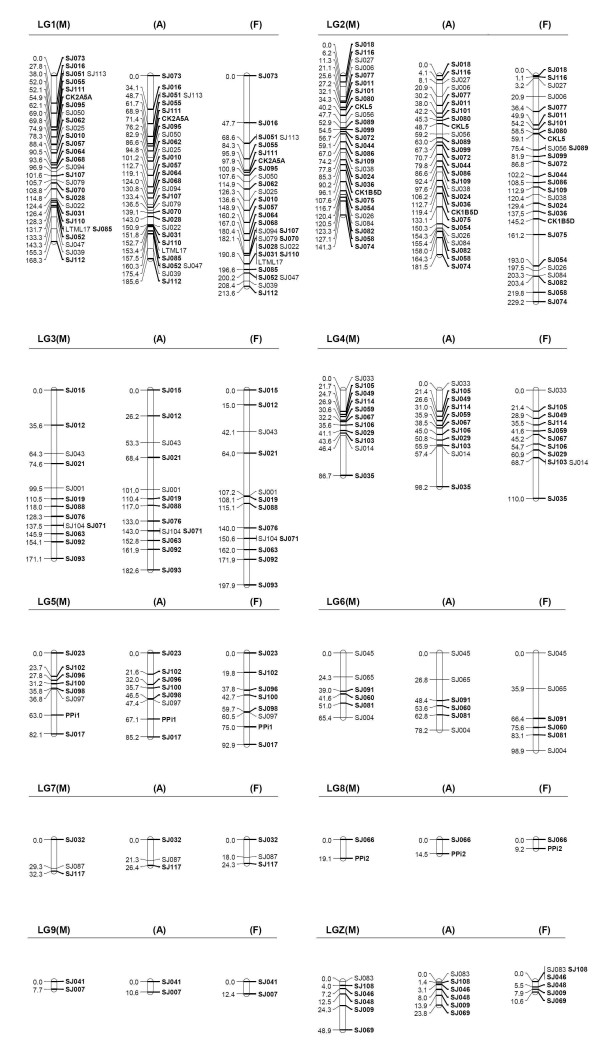
**The best-position linkage groups (male-specific, M; sex-average, A; and female-specific; F) in Kosambi centimorgans for the Siberian jay**. The markers in boldface font are framework loci with unambiguous relative position between each other.

**Figure 2:**
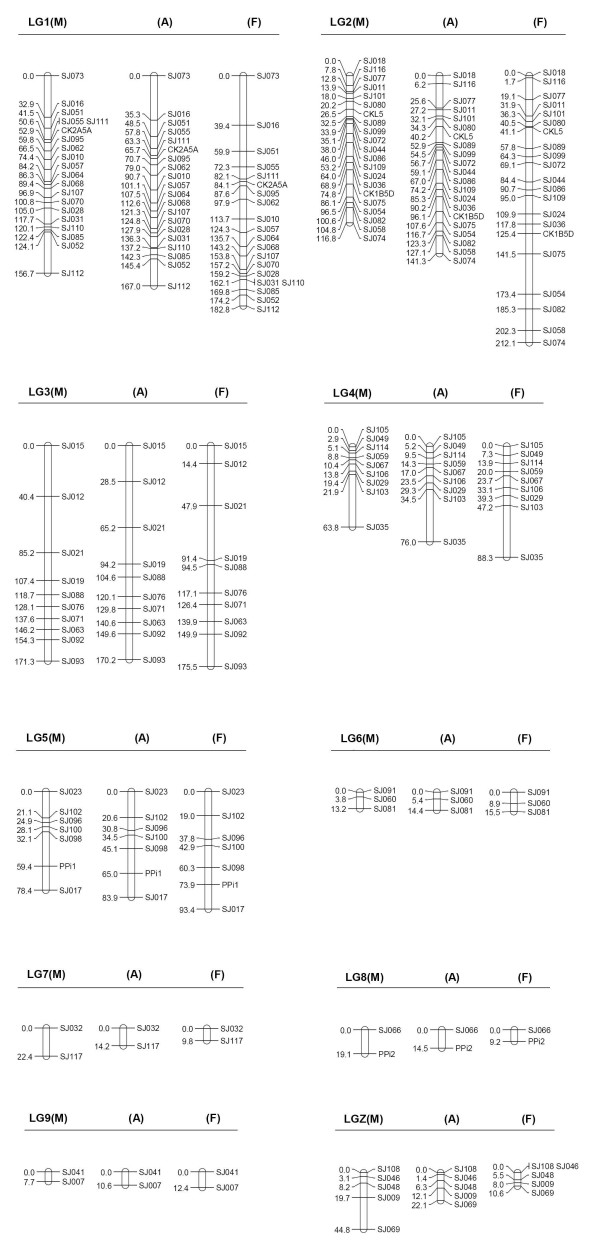
**The framework linkage groups (male-specific, M; sex-average, A; and female-specific; F) in Kosambi centimorgans for the Siberian jay**.

An overview of the linkage data is given in Figures 1, 2 and Table 1 depicting the best-position and framework linkage maps and their characteristics. Of the 117 microsatellites, 107 loci (91%) were assembled into 9 autosomal linkage groups (LG1 – LG9) and one Z-chromosome linkage group (LGZ), and the other 10 polymorphic microsatellites (SJ002, SJ003, SJ005, SJ020, SJ030, SJ034, SJ118, MJG1, PER1 and LTML8) appeared to be unlinked to any other marker by two-point analysis with LOD scores < 3.0. Of the ten unmapped markers, six had less than 60 informative meioses while four had more than 200 informative meioses. Of the nine autosomal LGs, seven comprised of six or more loci and the remaining three LGs each contained two or three loci, with an average of *ca*. 11 microsatellites per LG. For the sex-average autosomal LGs in the best-position map (Figure 1), the meiotic lengths, evaluated as the distance between the outermost markers on each LG, ranged from 10.6 cM (LG09) to 185.6 cM (LG01) spanning in total 862.8 cM of the Siberian jay genome. The average marker interval was 9.69 cM calculated as the arithmetic mean of the map distances between adjacent markers (Table 1). On the maps, 35.9% (33/92) of the intervals between markers varied from 0 to 5 cM, 31.5% (29/92) ranged from 5 to 10 cM, and 20.7% (19/92) from 10 to 20 cM, and 12% (11/92) were > 20 cM.

**Table 1:** Characteristics of the best-position and framework maps for Siberian jay

LG	Best-position map (cM)						Framework map (cM)					
	
	No. of loci	Sex average	♀	♂	Average inter-marker distance^*a*^	Ratio of ♀/♂ maps	No. of loci	Sex average	♀	♂	Average inter-marker distance^*a*^	Ratio of ♀/♂ maps
LG1	29	185.6	213.6	168.3	6.87	1.27	20	167.0	182.8	156.7	8.35	1.17
LG2	27	181.5	229.2	141.3	6.72	1.62	21	141.3	212.1	116.8	6.73	1.82
LG3	13	182.6	197.9	171.1	16.6	1.16	10	170.2	175.5	171.3	17.02	1.02
LG4	11	98.2	110	86.7	9.82	1.27	9	76.0	88.3	63.8	8.44	1.38
LG5	8	85.2	92.9	82.1	12.17	1.13	7	83.9	93.4	78.4	11.99	1.19
LG6	6	78.2	98.9	65.4	15.64	1.51	3	14.4	15.5	13.2	4.80	1.17
LG7	3	26.4	24.3	32.3	13.2	0.75	2	14.2	9.8	22.4	7.10	0.44
LG8	2	14.5	9.2	19.1	15.5	0.48	2	14.5	9.2	19.1	7.25	0.48
LG9	2	10.6	12.4	7.7	10.6	1.61	2	10.6	12.4	7.7	5.30	1.61
LGA^*b*^	101	862.8	988.4	774	9.69	1.28	76	692.1	799	649.4	9.11	1.23
LGZ	6	23.8	10.6	48.9	4.76	0.22	5	22.1	10.6	44.8	4.42	0.24

♀ Female-specific linkage groups

♂ Male-specific linkage groups

^*a *^The average inter-marker distance is based on the sex-average map

^*b *^total autosomal linkage groups

Sex-specific autosomal maps were also constructed (Figure 1). The sum of the length of all autosomal LGs was 774.0 cM in males and 988.4 cM in females, with an average intermarker spacing of 8.6 cM and 12.1 cM, respectively. The male map comprises LGs ranging in length from 7.7 cM to 171.1 cM while the female map contains LGs with a length from 9.2 cM to 229.2 cM (Table 1). Out of the nine pairs of male and female LGs, seven were larger in the female map and two were larger in the male map. In total, the autosomal LGs were smaller in males as compared to females with a female-to-male map ratio of 1.28. The sex-average map was intermediate in length between sex-specific maps, and 1.12 times longer than the male map.

Framework markers, which could be ordered with LOD score of 3.0 or greater (indicating odds of 1000:1), are indicated in bold fonts in the best-position map (Figure 1). Of the total 107 mapped markers, 81 loci were significantly ordered in the framework map and most (16/24) of the remaining loci could be placed with significant support in either of two alternative intervals. When only the framework markers were considered, the total size of autosomal linkage groups was 692.1 cM in the sex-average map, 649.4 cM in the male map, and 799 cM in the female map (Figure 2 and Table 1). We compared recombination distances between adjacent framework markers that were mapped on both the best-position map and the framework map and overall the female-to-male ratio for the framework map was 1.23, slightly lower than the ratio of 1.28 observed above in the best-position map.

Among the Z-linked microsatellites, all six markers showed highly significant linkage between each other with LODs > 37.0. This linkage group spanned 23.8 cM in the sex-average map, 48.9 cM in the male map and 10.6 cM in female map, which corresponds to a female-to-male ratio of 0.22. The female linkage map indicates the position and approximate extent of a pseudoautosomal region (PAR) from SJ069 to beyond SJ048. In females, no recombination was observed between SJ083 and SJ046 and no heterozygosity was observed for these markers; therefore they must lie outside the pseudoautosomal region, suggesting that the pseudoautosomal boundary lies between SJ048 and SJ046. As expected, there is considerable difference in male and female recombination rates for the pseudoautosomal region of the Z-chromosome, with the male distance between SJ108 and SJ069 being 41.7 cM as compared to the female distance of 5.1 cM. This increased sex-specific recombination rate between the three pseudoautosomal loci was similar to the observations in the pseudoautosomal regions of the mammalian sex chromosomes, for example in humans [50], ovines [51] and bovines [52]. In the sex-average map, the map density was 4.0 cM/marker among all Z-linked markers and 3.3 cM/marker among the five framework loci.

### Differences in recombination rate between sexes

**Figure 3:**
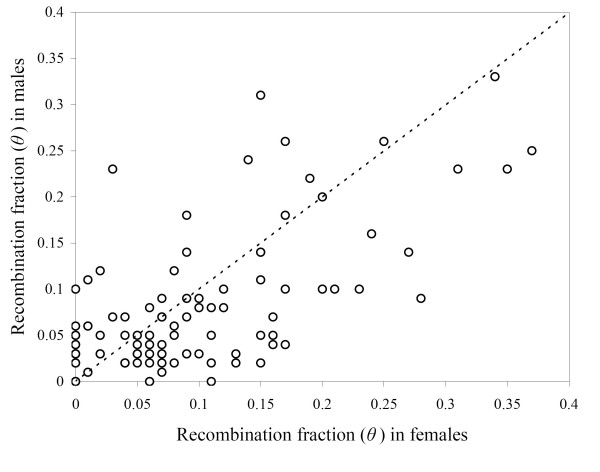
**Female *vs*. male recombination fraction for 97 pairs of adjacent markers from the 10 linkage groups in the Siberian jay**.

In addition to a much shorter total length of autosomal linkage maps in males than in females, the maps allowed comparison of meiotic recombination rate between sexes. The sexes show significant differences in recombination rates, both in general and for specific pairs of linked markers (Wilcoxon's signed-rank test, *P *= 0.037; Figure 3 and Table 2). In the best-position map (Figure 1), the proportion of intervals in the autosomal linkage groups that demonstrated a higher recombination fraction in females was 54.3%. Among all adjacent autosomal markers the recombination fraction was 1.28 times higher in females than in the males. However, there were exceptions to this in some LGs, and in some specific intervals within LGs. For instance, LG7 and LG8 exhibited higher recombination fractions in males than in females (Table 2). The number of intervals that show higher recombination fractions in the male map relative to the female map was less, but not negligible (Figure 3). When investigating the distortions over the autosomal linkage groups, two of the nine linkage groups showed significant (*P *< 0.05) difference in recombination rate between the sex-specific maps (Table 2). This was also observed when the overall map length was investigated for sex-specific difference. All in all, these results suggest that overall recombination is significantly suppressed in male meiosis as compared to female meiosis.

**Table 2:** The Wilcoxon's signed-rank test results for recombination fraction (*θ*) between sexes with linkage groups

Linkage groups	*N*^*a*^	Average *θ*_F_^*b*^	Average *θ*_M_^*c*^	*θ*_F_/*θ*_M_	Wilcoxon's signed-rank test (*P*)^*d*^
LG1	28	0.071	0.055	1.29	0.174
LG2	26	0.085	0.054	1.57	0.046 *
LG3	12	0.152	0.133	1.14	0.323
LG4	10	0.101	0.077	1.31	0.038 *
LG5	7	0.127	0.110	1.15	0.687
LG6	5	0.178	0.126	1.41	0.192
LG7	2	0.115	0.145	0.79	0.5
LG8	1	0.09	0.18	0.5	-
LG9	1	0.12	0.08	1.5	-
Total autosomal	92	0.101	0.079	1.28	0.037 *
LGZ	5	0.02	0.094	0.21	0.025*

^*a *^number of intervals; ^*b *^average recombination fraction in females; ^*c *^average recombination fractions in males; ^d ^*, significant, *P *< 0.05.

### Comparative mapping

**Figure 4:**
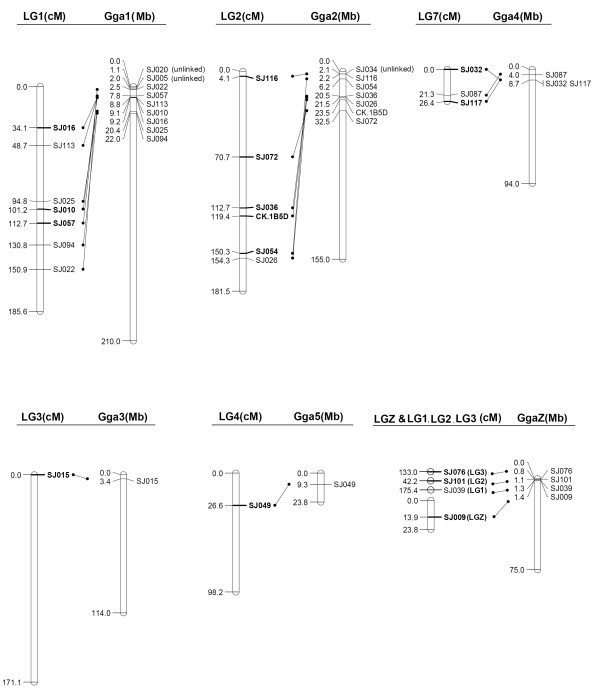
**Comparison of the whole of all sex-average linkage groups in the Siberian jay and the whole of chicken chromosomes**. The homologous loci on linkage groups and chromosomes are presented with their genetic positions (cM) on the best-position map or their genomic locations (Mb) in the chicken genome. Siberian Jay marker names in bold font indicate framework loci.

The BLAST searches under both settings generated the same set of significant hits at 1e^-10^. We found 25 homologous (21.4%, 25/117) zebra finch sequences using a cross-species MEGABLAST search in NCBI's zebra finch genome database (Table 3). By BLAST searching using two methods, in total 22 mapped (20.5%, 22/107) and three unlinked loci for which a homologous sequence could be identified in chicken were assigned to a chromosomal location in the chicken genome (Table 3). The 10 Siberian jay LGs corresponded to five different autosomal and one Z chromosome in chicken. Loci from the same Siberian jay LG matched sequences on a single chicken chromosome in the BLAST analysis (Figure 4 and Table 3), with the exception of loci SJ039, SJ101 and SJ076 on the autosomal LGs (LG1, LG2, and LG3, respectively) that mapped to chicken chromosome Z (GgaZ), whereas the other loci on these LGs mapped to the chicken autosomes. Likewise, most loci on the same chicken chromosome matched sequences on a single Siberian jay LG, with the exceptions of three unlinked loci (SJ005, SJ020 and SJ034) and loci SJ009, SJ039, SJ101 and SJ076 on GgaZ that mapped to four different LGs, LGZ and LG1, LG2, and LG3, respectively.

**Table 3:** The homologous loci of microsatellites mapped in Siberian jay on the chicken and zebra finch genome assigned using BLAST analyses of the clone sequences of the microsatellites and the homologous zebra finch WGS sequences.

Locus	Linkage group	Zebra finch Ensemble ID	Chicken Ensemble ID	Gga ^*a*^	Chicken genome start position (bp)
SJ010	LG1	gb|AC188472.1	NW_001471554.1 Gga1_WGA51_2^*b*^	1	9,129,918
SJ016	LG1	-	NW_001471545.1 Gga1_WGA43_2^*b*^	1	9,256,107
SJ022	LG1	-	NW_001471526.1 Gga1_WGA26_2^*b*^	1	2,462,620
SJ025	LG1	gb|AC188472.1	NW_001471534.1 Gga1_WGA33_2^*b*^	1	20,407,077
SJ057	LG1	-	NW_001471554.1 Gga1_WGA51_2^*b*^	1	7,801,901
SJ094	LG1	-	NW_001471554.1 Gga1_WGA51_2^*b*^	1	22,042,793
SJ113	LG1	-	NW_001471545.1 Gga1_WGA43_2^*b*^	1	8,757,200
SJ020	unlinked	-	NW_001471529.1 Gga1_WGA29_2^*b*^	1	1,098,266
SJ005	unlinked	gb|AC188184.3	NW_001471513.1 Gga1_WGA14_2^*c*^	1	2,080,202
SJ036	LG2	-	NW_001471639.1 Gga2_WGA66_2^*b*^	2	20,469,429
SJ072	LG2	-	NW_001471633.1 Gga2_WGA60_2^*b*^	2	32,273,874
CK.1B5D	LG2	gb|AC225878.2	NW_001471639.1 Gga2_WGA66_2^*b*^	2	23,539,833
SJ026	LG2	gb|AC206427.2	NW_001471639.1 Gga2_WGA66_2^*c*^	2	21,465,630
SJ054	LG2	gb|AC188186.2	NW_001471633.1 Gga2_WGA60_2^*c*^	2	6,229,425
SJ116	LG2	gb|AC148379.2	NW_001471633.1 Gga2_WGA60_2^*c*^	2	22,279,892
SJ034	unlinked	-	NW_001471654.1 Gga2_WGA81_2^*b*^	2	2,075,856
SJ015	LG3	gb|AC188188.2	NW_001471673.1 Gga3_WGA106_2^*c*^	3	3,458,567
SJ032	LG7	-	NW_001471681.1 Gga4_WGA107_2^*b*^	4	8,679,773
SJ087	LG7	gb|AC155211.2	NW_001471681.1 Gga4_WGA107_2^*c*^	4	4,015,500
SJ117	LG7	-	NW_001471681.1 Gga4_WGA107_2^*b*^	4	8,679,607
SJ049	LG4	-	NW_001471710.1 Gga5_WGA136_2^*b*^	5	9,314,084
SJ009	LGZ	-	NW_001488876.1 GgaZ_WGA457_2^*b*^	Z	1,412,682
SJ101	LG2	gb|AC213969.2	NW_001488849.1 GgaZ_WGA430_2^*b*^	Z	1,097,987
SJ039	LG1	gb|AC231254.2	NW_001488882.1 GgaZ_WGA463_2^*c*^	Z	1,315,022
SJ076	LG3	gb|AC188376.1	NW_001488862.1 GgaZ_WGA443_2^*c*^	Z	825,441
SJ050	LG1	gb|AC189030.1	-	-	-
SJ051	LG1	gb|AC189030.1	-	-	-
SJ055	LG1	gb|AC189030.1	-	-	-
SJ064	LG1	gb|AC206426.2	-	-	-
SJ112	LG1	gb|AC192320.2	-	-	-
CK.2A5A	LG1	gb|AC188469.1	-	-	-
SJ029	LG4	gb|AC188184.3	-	-	-
SJ033	LG4	gb|AC188469.1	-	-	-
SJ106	LG4	gb|AC188184.3	-	-	-
SJ017	LG5	gb|AC192320.2	-	-	-
SJ041	LG9	gb|AC210531.1	-	-	-
SJ069	LGZ	gb|AC188466.2	-	-	-
SJ083	LGZ	gb|AC229626.2	-	-	-

^*a *^Chicken chromosome number; ^*b *^Via the clone sequences of the Siberian jay microsatellites; ^*c *^Via the homologous zebra finch WGS sequences

The relative order of the markers mapped to the Siberian jay LGs was compared with the same loci on chicken chromosomes in Figure 4. The chicken-Siberian jay comparison indicated that the order of loci was strikingly different between the chicken macrochromosomes Gga1, Gga2 and Siberian jay LG1, LG2 in the best-position map, respectively. Although some loci shared the same relative order in the two species, large rearrangements of the chromosome would have been necessary to give rise to the different orders found here. The cases of inter- and intra-chromosomal rearrangements were involved mostly in the framework loci and represented a large proportion of Gga-LG1 that spanned around a 120-cM Siberian jay/20-Mb chicken interval. The three unlinked loci, SJ005, SJ020, and SJ034, were located at the distal ends of two chicken macrochromosomes, Gga1 and Gga2.

## Discussion

This study constitutes the first mapping effort of the Siberian jay genome, and is among the first ones to present a preliminary linkage map for any entirely natural vertebrate species from the wild (reviewed in [28]). The linkage map was composed using 107 polymorphic microsatellite loci typed on *ca. *350 animals, making it one of the most detailed linkage maps available for natural animal populations [28]. In fact, it is one of only a few genetic linkage maps of wild bird species to date. Apart from the revealing evidence for sex differences in recombination rates, the constructed maps represent an excellent resource from which the markers may be selected for future mapping projects in this and related species, as well as for comparative genomic studies of genome organisation. In what follows, we will discuss the salient features of the constructed linkage maps in comparison to similar maps and results from earlier studies. In particular, we will pay attention to sex-specific differences in recombination rates, map coverage and some other issues deserving future attention.

### Genotyping in the mapping population

The constructed map contains 107 microsatellites, of which 101 are autosomal, three Z-chromosome-specific and three pseudoautosomal loci (see below). The ideal set of molecular marker data for linkage mapping has no missing values, no genotyping errors and the markers segregate in the expected ratio for the specific type of population [53]. In practice, however, mapping data is compromised in all of these respects. However, as simulated and concluded in previous research [53], the effect of missing genotypes depends greatly on the sample size: the smaller the sample, the more severe the effects are likely to be. In comparison to published simulations, in which the results were found to be quite robust with 150 individuals and 10% of missing values, our data had much less missing values (4.5%) and a larger sample size (349 individuals). Hence, the potential biases in our map due to missing values are unlikely to be severe.

### Map construction and genome coverage

The resulting linkage map of 107 microsatellites spans a total sex-average length of 886.6 cM, with 10 LGs. The number of markers within the data that showed significant linkage (LOD > 3.0) with at least one other marker was very high (107/117, 91.5%), a phenomenon observed also in a linkage mapping study of the great reed warbler [53]. Of the 10 unlinked markers (LOD < 3.0), four demonstrate sufficiently informative meiosis, suggesting that these four unlinked markers were most likely located on unique chromosomes or chromosome arms. The haploid number of *ca. *40 chromosomes is typical for passerine birds (e.g. 7 macro-, 32 micro-, and a pair of sex-chromosomes in the zebra finch genome [54]) and the chicken genome is composed of 39 haploid chromosomes (8 macro and 30 micro, and a pair of sex chromosomes [54]). Thus, the presence of small groups and unlinked markers indicates that appreciable gaps of at least 29 additional linkage groups should be filled to consolidate the Siberian jay map. The discrepancy between the number of LGs and the haploid number of chromosomes has been commonly reported when constructing linkage maps in avian species (see [4]). These results could be explained in terms of the non-random distribution of microsatellites in the avian genome, where microchromosomes – typically scarce of microsatellites [55] – constitute a large proportion (*ca*. 80%) of the total number of chromosomes.

Considering the avian order Passeriformes, the current sex-pooled map of the Siberian jay at 887 cM is larger than recent maps in the great reed warbler (155 – 237 cM [4]; 707 – 858 cM [6]), but smaller than those in the collared flycatcher (1787 cM [7]) and the zebra finch (1068 cM [8]). The maximum genome coverage of the markers described in this study was estimated to be 1187 cM (887 + 200 + 100 cM) (see[56,57]), covering about a third of the Siberian jay genome of ~3800 cM, if estimated by assuming a similar genome size as in chicken [54]. It is clear that some of the microchromosomes are poorly represented, or not represented at all, in the current map. This poorer coverage of the microchromosomes, as well as of the Z-chromosome, is in good agreement with the observations in chicken [56], Japanese quail [58], duck [3], and great reed warbler [4,6]. However, it was argued in [8] that for the whole-genome map of the zebra finch using 876 SNPs the shorter length of linkage map relative to that of the chicken is ascribed to its unusually lower recombination rates. Similarly, lower recombination rates and smaller length of linkage map were also revealed in the collared flycatchers based on 147 gene markers and 64 microsatellites [7]. Since it has been suggested that passerines generally have lower recombination rates than the chicken [8], the small map sizes here may also be partly due to reduced recombination rates in the Siberian jays. Nevertheless, the first-generation linkage map will undoubtedly evolve as more markers are added, with some additional linkage groups forming and some pairs of now described linkage groups coalescing into a single group. As linkage maps continue to develop, future work on the genetic map will increase the genome coverage by adding more novel genetic markers, for example, 1000's of SNP loci and additional markers will improve the resolving power of the map. A more saturated map should give more information about the genome size, exact karyotype number and chromosomal rearrangement of Siberian jay.

The constructed framework map was comprised only of loci with unambiguous positions relative to each other. As shown in Table 2, only a slight expansion of the framework map size was detected when the non-framework markers were included in the analysis. Non-significant (*P *> 0.05) difference in the interval distances between framework markers was found in the sex-specific maps when non-framework markers were included or not, which can be due to the fact that most (19/26) of the non-framework loci still can be placed with significant (LOD = 2; odds of 100:1) support in either of two alternative intervals at LOD = 3 (odds of 1000:1). However, on the sex-average maps, a significant (*P *< 0.05) difference in distances between framework markers was revealed when including or excluding the non-framework markers from the analysis. This is probably due to the overall effect of the recombination heterogeneity between the sexes on estimates of sex-average map distances between framework and non-framework markers [49]. Thus, our results indicate that the framework maps can serve as the backbone of the best-position maps with a high level of confidence. Moreover, the framework map should also therefore provide a robust basis with which to apply to other pedigrees and for comparing genomic rearrangements with closely related species.

### Z-chromosome

By assigning six genetic markers to the Z-chromosome on the basis of Mendelian inheritance and additional two-point analysis tests, we found that the recombination rates in the Z-chromosome linkage group differed substantially among the sexes. In particular the ratio of recombination fraction in females (*θ*_F_) and males (*θ*_M_), *θ*_F_/*θ*_M_, in the LGZ (*θ*_F_/*θ*_M _< 1) contrasted markedly with those in seven of the nine autosomal linkage groups (*θ*_F_/*θ*_M _> 1). These results conform to the Z-chromosome linkage results from the chicken [56] and the great reed warbler [4,6], suggesting significant sex-specific heterogeneity in recombination rate on the avian Z-chromosome. Between markers SJ046 and SJ069, a small amount of recombination (recombination fraction *θ *= 0.02–0.05) was found to occur between Z and W chromosomes in the mapping pedigree. Z-W recombination has only once been previously reported in previous mapping efforts of birds based on SNPs (see [8]), but equivalent X-Y recombination has been observed in linkage mapping studies of sex chromosomes in many other species such as in salmonid fishes [59], rainbow trout [60], cattle [61], sheep [51,62], oyster [63], and humans [64]. However, in contrast to increased recombination rates in heterogametic sex (male, XY) as observed in the pseudoautosomal regions of above mentioned studies, an overall 8-fold lower recombination rates between SJ048 and SJ069 were found in female (ZW) as compared to male (ZZ) Siberian jays. In addition, it seems as the pseudoautosomal region is extensive in Siberian jay (15.8 cM/23.8 cM, 66.4%). While in other species, 13.3% (20.1 cM/151 cM) were reported for the PAR in the bovine sex-average consensus map [52], 6.3% (3.5 cM/55.8 cM) in the male-specific and 45.1% (54.2 cM/121 cM) in the female-specific map of the sex chromosomes in sheep [51], and a very small proportion of PAR in the chicken sex chromosomal maps [56]. It is unclear why these are so, but potential explanations include the evolutionary strata [65], patterns of genome variability [66], distribution of sex-biased loci [67,68], chiasma interference [69] and selection [70] on the avian Z-chromosome. Since the difference was found in the recombination rates and the change of genome structure during the avian evolution between Siberian jay and other wild bird species, we would like to speculate that the pattern of genome variability and distribution of sex-biased loci may have contributed more to this observation. Further investigations using more pseudoautosomal markers are needed to verify this result and understand its origin and significance.

### Recombination heterogeneity

When testing for sex-specific differences in recombination rates in different linkage groups and over the total autosomal map, we found evidence for statistically significant distortions towards reduced male recombination fractions only in two autosomal LGs, rather than in each of the nine autosomal LGs. This indicates that the sex-related differences in recombination rates are confined to certain, specific parts of the genome. This result is not unexpected given that in many species there is a large variation in the recombination rates within and among chromosomes (see [71,72]). Similar situations of linkage group sex-specific distortion have also been observed in other species for example in marsupials (e.g. [73,74]) and in various aquatic organisms (the pacific oyster [63]; the tiger pufferfish [75]; and the turbot [76]).

Sex-specific differences in recombination rates have been found in a diverse range of organisms from molluscs (e.g. [77]) and fish (e.g. [49]) to mammals (e.g. pig [78]; cattle [79]; and human [80]). However, in birds various patterns have been reported: little evidence of heterochiasmy in chicken (*Gallus domesticus*, [54]) and zebra finch (*Taeniopygia guttata *[8]); higher rates of recombination in males than in females in linkage maps of blue tit (*Parus caeruleus *[81]) and collared flycatcher (*Ficedula albicollis *[7]); and higher rates of recombination in females than in males in maps of great tit (*Parus major *[81]), great reed warbler (*Acrocephalus arundinaceus *[4,6]) and house sparrow (*Passer domesticus *[14]). The sex-bias (females > males) observed in Siberian jay conforms to the last pattern opposing the Haldane's prediction [82] according to which the heterogametic sex should show lower recombination rates than the homogametic sex. So far the comparative data suggest a divergence between the genetic maps of the chicken (higher recombination rate and little difference in recombination rate between sexes) and the passerines (lower recombination rate and significant difference in recombination rate between sexes). Interestingly, on the one hand, we note the pronouncedly different patterns of recombination observed in collared flycatcher [11], zebra finch [8] and Siberian jay here, all of which are passerine birds. It has been proposed that heterochiasmy is the result of sexual selection, with the sex experiencing the greater variance in reproductive success exhibiting the lower recombination rate [83]. However, this explanation may not be relevant here because both collared flycatchers [84] and zebra finches [85] are polygamous, whereas the Siberian jay is a monogamous species [34,37], so that the reproductive success should be very similar between the sexes. On the other hand, we found that the female:male recombination rate (1.21) in the best-order map was much smaller than that observed in the great reed warbler where it varied from 2.15 (microsatellites[4]) to 1.86 (AFLP markers [6]). This difference is not necessarily biologically meaningful as it could be attributable to less informative and smaller number of loci (albeit larger number of individuals) in the earlier studies. Furthermore, since marker density in both Siberian jay and great reed warbler maps is relatively low and the sex-specific recombination rates heterogenous among and within linkage groups, the estimated female:male recombination rates among the maps may vary if different genomic regions are mapped

The observed sex-specific recombination rates are potentially influenced by numerous factors, and at the moment, there is no consensus in respect to the relative importance of mechanisms accounting for the recombination differences (see [63,73]). For example, numbers of hypotheses including sexual selection [83]), haploid selection [86], sex differences in the internal or external environment [4] and sex differences in gene expression [67] have been evoked to explain the heterochiasmy pattern in birds. In this long-term isolated population studied here, we assume that the heterochiasmy could be more or less attributed to the sexual selection and the sex differences in the internal environment. However, irrespectively of the proximate mechanisms, the significant sex differences in recombination rates in the Siberian jay have obvious practical implications for future work. For instance, the lower average rate of recombination in males than in females should be advantageous for QTL-mapping of genetic traits in initial low resolution analysis [87].

### Conserved synteny, but changed marker order, in a genomic comparison with the chicken

The study confirms the remarkable degree of conserved synteny between passerines and chicken (see [4,6-9,13,14]), albeit based on only a small number of comparable chromosomes. Surprisingly, loci SJ039, SJ076 and SJ101 on autosomal LGs were mapped to GgaZ, which was known to be homologous to Z chromosome in passerines [this study, [8,9,13]]. As argued in [12], this observation may suggest chromosome fusions/fissions, a spurious match, or a more complex history of the loci. Future mapping studies may help to elucidate this. Locus SJ009, which was identified within the PAR region for the Siberian jay, was found to be conserved between LGZ and GgaZ. This is consistent with previous reports of cytogenetic and genetic mapping of the Z chromosome that the PAR region was conserved between chicken and passerines [8,88]. However, this is not the case for the other three loci (SJ039, SJ076 and SJ101) on the autosomal LGs in Siberian jay. Thus, there are more inconsistencies than consistencies in the Z-chromosomal genomic comparison between Siberian jay and chicken in this study, which is different from previous results of Z-chromosomal synteny between the passerines and chicken [9,12,13]. Further studies to explore the potential explanations are needed in the future. The BLAST analysis located three unlinked loci, SJ005, SJ020 and SJ034 on Gga1 and Gga2. This can be explained by the fact that these three markers had few informative meioses and therefore low power in linkage analysis and/or that these loci are located in telomeric region, which may have a higher recombination rate. Indeed, as indicated in Figure 4, these three loci are located to the end of Gga1 and Gga2 in chicken (SJ005 at 2.0 Mb of Gga1, SJ020 at 1.1 Mb of Gga1; SJ034 at 2.1 Mb of Gga2).

Although synteny was conserved, there were multiple cases of inter- and intra-chromosomal rearrangements. Similar patterns have also been observed in other passerine birds on both sex and autosomal chromosomes (see [9,14]). As a contrast to the previous studies, the extent of chromosomal rearrangements as observed in the Gga1-LG1 and Gga2-LG2 comparisons has not previously been reported. For example, in the comparative mapping analysis between the zebra finch and the chicken, only a few instances of inversions and translocations were found for chromosome 1 [8]. Of the total 22 homologous loci between chicken and Siberian jay found here, 12 loci (12/22, 55%) were involved in inter- or intra-chromosomal rearrangement, while a lesser proportion of the rearrangements were observed in the chicken/collared flycatcher (26/159, 16.4%) [7] and chicken/great reed warbler (7/44, 15.9%) comparisons [13]. There are several possible explanations for the different rates of genome evolution, e.g. the species-specific differences in rate of mutations and/or genome evolution could have contributed to the more or less genetic similarity of different passerines to the chicken; also, it has been suggested that population structuring in species with otherwise large population sizes facilitate chromosomal rearrangements. However, the scenario outlined here should be considered with caution because (*i*) a small number of comparable chromosomes (*n *= 6) was identified here between the Siberian jay and the chicken; (*ii*) most of the chromosomal rearrangements are only from three chromosomes (chromosomes 1, 2 and Z). Thus, further extensive comparative mapping of genomes and genetic linkage maps of the chicken, zebra finch, great reed warbler and Siberian jay with more and denser genome coverage should provide a more detailed picture of marker order rearrangement between passerines and chicken during avian evolution. Also, more sequence data to make the genome comparison between the chicken and the zebra finch, whose genomes are only currently sequenced in birds, is worthwhile. At any rate, the important message from our comparative analyses is that a high level of observed synteny does not necessarily mean conserved marker order. If the internal rearrangements tend to occur frequently across the genome, then map information derived from one species will not be readily transferable to another.

## Conclusion

A salient feature of genetic linkage maps is that they improve with use. The described linkage maps of Siberian jay genome presented here, based on a large number of informative meioses, will serve as a basis for a future high-resolution map. In particular, the framework map should enable the rapid construction of the next generation of higher-density linkage maps. From the evolutionary inference point-of-view, the linkage maps constructed here have potential to contribute to our understanding of patterns of genome evolution in birds, and that of passerines in particular. In addition, despite the need for increased density of markers, the maps will be also potentially useful for identifying QTLs and genomic regions related to traits of ecological importance. Furthermore, because the maps are constructed with codominant markers, they should be transportable to other pedigrees in this species, as perhaps also to closely related species where the microsatellites are conserved. Hence, the map will serve as a reference map for genomic analyses in the Siberian jay, as well as a useful resource for comparative genomic studies in birds. Last but not least, given the access to individual level fitness data from this pedigreed population, the map might prove useful for more detailed studies in genetics of fitness and inbreeding depression in this small isolate population [42].

## Methods

### Study species and pedigree

The Siberian jay (*Perisoreus infaustus*) is a medium sized (body mass *ca*. 85–90 g) and relatively long-lived (average generation time *ca*. 4 years) oscine passerine bird from the Corvidae family [89,90]. It has a wide geographical distribution range in northern Eurasia where it occurs in the taiga forests from Fennoscandia to Siberia [40,91]. The Siberian jay has a stable socially monogamous breeding system and a prolonged brood care in which the dispersal of the young is delayed [34,37]. Hence, it lives in small territorial groups consisting of a breeding (alpha) pair, their retained offspring and/or unrelated immigrants [34,39,92]. Genetic diversity and structuring of the species has been investigated with mitochondrial DNA sequence analyses [91] and with a few available microsatellite markers [40,93]. However, its karyotype and genome structure remain undescribed. So far, 2n = 80 chromosomes is common in the order Passeriformes (e.g. [13,88,94,95]. Likewise, as in all avian species with heteromorphic sex chromosomes, the sex-determination in the Siberian jays conforms to the ZW female-ZZ male system [96].

The material for this study comes from a Siberian jay population breeding at Suupohja (*ca. *66°18'N, 29°29'E), western Finland. Annual individual-level monitoring of the population began in 1974 and has continued ever since. More details concerning the study population and field monitoring activities can be found from [40,42,93,97]. To obtain DNA, all animals used in this study were sampled for approximately 200 μl of blood from the wing vein, or for tail feathers. Pedigrees for the population were established through direct field observation in combination with molecular parentage verification using microsatellite markers [40,93,97].

The mapping population analysed here was selected from a large Siberian jay data set comprising more than 1000 individuals. Five major families consisting of 349 animals (172 males and 177 females) from the years 1975 to 2006 were identified. In the total pedigree, 169 different male and 142 different female offspring were fathered by 85 males and mothered by 95 females. The five family pedigrees used for linkage analysis varied in depth from four to six generations and ranged in size from 72 to 97 animals. These family pedigree configurations yield large numbers of pairwise relationships, which include, but are not limited to, parent-offspring, full-sibs, half-sibs, grandparent-grandchild, great grandparent-great grandchild, avuncular, half-avuncular, and the first cousin relationships. The complex nature of the pedigree is mainly ascribed to two properties of the species. One is that the Siberian jays are philopatric, and some of the young remain in their natal territories up to the age of three years [98], such that the offspring occasionally enter the pedigree as parents in subsequent years. The other is that a few birds in one family are also involved as parental animals in another family and that several birds are present in more than one family pedigree. A more thorough description of the structure of the mapping pedigree can be found from [40,42,93].

### Microsatellite markers and genotyping

Genomic DNAs were extracted from blood samples or from the tail feather shafts using the DNeasy Blood & Tissue Kit (Qiagen, Helsinki, Finland) following the manufacturer's instructions as described earlier in [44]. A total of 117 polymorphic microsatellite loci were used, including nine markers previously described in [93], 92 loci in [44] and 16 developed in this study. Development of the 16 new microsatellites reported in this study was carried out as described in [44] and sequence data from the loci have been deposited with EMBL/GenBank Data Libraries (see Additional File 1). Detailed information of all these loci including their names, primer sequences, Genbank accession numbers, repeat motifs and the size range of the detected alleles is available in Additional File 1. All PCRs were multiplexed by combining 3 – 4 primer pairs in each reaction. The amplifications were performed in 10 μl reactions with 2 pm of each primer, 1× Quiagen multiplex mastermix, 0.5 × Q-solution, and approximately 30 ng of template DNA. The general temperature profile was 15 min at 95°C, 30 cycles of 30 sec at 94°C, 90 sec at 56°C and 60 sec at 72°C, followed by a final extension step at 72°C for 10 min. PCR products were analysed on MegaBACE™ 1000 capillary sequencer (GE Healthcare Life Sciences, Little Chalfont, UK) and genotypes were determined using program Fragment Profiler version 1.2 (GE Healthcare Life Sciences, Little Chalfont, UK). In addition to the studied samples, one negative control and a reference DNA were included in all the amplifications in order to exclude the contaminant in the solutions and standardize the size of allele fragments of samples from different plates, respectively.

### Genotype cleaning

Identification and elimination of genotyping errors is a critically important task in genome mapping projects (see [53]). Several strategies have been applied to identify and eliminate any latent genotyping errors in this study. Initially, all individual genotypes were checked manually before export to Microsoft Excel worksheets. In addition, genotypes were analysed for Mendelian inheritance in the known pedigree using the "PREPARE" option in CRIMAP program version 2.4 [99] and incompatible genotypes were blanked. These blanks were either refilled with the correct genotypes after rechecking within Fragment Profiler or after running new PCRs where necessary. If these 'refill's did not work out, genotypes were scored as missing. In some cases, genotypes that were consistent with the Mendelian segregation, but highly improbable due to low relative allele frequencies in the pedigree, were also eliminated. Moreover, after preliminary alignment, the CHROMPIC option of the CRIMAP was used to identify unlikely double crossovers. Data contributing to double crossovers were re-examined and, if suspect, regenerated for second analysis. The overall genotype call rate was 95.5% and when possible, missing data (mostly due to missing DNA samples) were inferred from the genotypes of parents/offspring.

### Linkage analysis and microsatellite characterization

Genetic linkage maps were constructed using the program CRIMAP version 2.4 [99]. In the analyses, autosomal and Z-linked loci were evaluated separately. First, the TWOPOINT option of CRIMAP was used to obtain an estimated recombination fraction and a logarithm of the odds (LOD) score for every pair of markers. A widely used two-point LOD score threshold of three was set as a criterion for significant linkage and was used for configuration of a linkage group. Then linked markers were ordered within group following a procedure similar to that employed in [6]. For multipoint analysis of larger linkage groups we used the option BUILD to select loci to be used as a framework for the continuing ordering of them, beginning with the most informative pair of markers and positioning additional markers one at a time in order of decreasing informativeness; the order of selected markers, also called framework loci, was supported by a LOD score of three or higher. The remaining markers were added to the framework map by lowering the LOD threshold value to 2.0 or 1.0 and were represented as accessory markers in their most likely or best position. Finally, the options FLIPS *n *and FIXED were used to evaluate the statistical support of the proposed order and the distance between markers, respectively. With the FLIPS *n *(*n *= 3 – 6) option, it is ensured that the odds in favour of the final order of each set of three to six markers were at least 1000:1 over alternative orders. Sex-average and sex-specific linkage genetic distances were estimated for each linkage group. Relative mapping distance was further tested within group using the FIXED option. All map distances are expressed as Kosambi centiMorgans (cM).

The measures of microsatellite variability, including the observed number of alleles (*A*_E_), observed heterozygosity (*H*_O_), Nei's [100] unbiased estimates of expected heterozygosity (*H*_E_) and polymorphic information content (PIC), were estimated with the Excel Microsatellite Toolkit 3.1 [101].

### Comparative analysis

Comparisons of clone sequences of the microsatellites in Siberian jay with the chicken genome sequence  were conducted using MEGABLAST . The orthologous zebra finch sequence was identified by performing another cross-species MEGABLAST search in NCBI's zebra finch genome resource . Further, the homologous zebra finch sequences identified above (including their flanks) were matched against the chicken genome assembly using the same method as the BLAST analysis between the microsatellites in Siberian jay and the chicken genome sequence. The searches were performed following a procedure as detailed in [7,12,13]. At the Expect value E = 1e^-5^, initial searches were performed under the default setting and later searches were under a less stringent set of parameters (the so called 'the relaxed setting' in [12]). A locus was assigned to a specific location in the chicken genome if (i) it provided a unique hit at E ≤ 1e^-10^, or (ii) it provided multiple hits at E ≤ 1e^-10 ^and within the hits the best hit had an E value at least 10 decimal places lower than the next best hit (see [12]). All the graphic maps were generated using MapChart version 2.2 [102].

## Authors' contributions

SJ designed the study, conducted the laboratory work and prepared the data. MHL inspected the data, performed the data analysis and wrote the manuscript. JM planned and coordinated the whole study, and contributed to the manuscript writing. All authors read and approved the final manuscript.

## Supplementary Material

Additional file 1 **Summary information on the Siberian jay microsatellites genotyped in this study.** Excel worksheet containing locus name, genbank accession number, primer sequences, allele size range, position and interval distance on linkage group, number of alleles, observed and expected heterozygosity, polymorphic information content, and references. Click here for file
